# Flexor Digitorum Profundus Avulsion With Distal Interphalangeal Joint Dorsal Dislocation: A Case Report

**DOI:** 10.7759/cureus.68444

**Published:** 2024-09-02

**Authors:** Swaroop Solunke, Ashwin Deshmukh, Abhishek Nair, Shubhankar Chopra, Archit Gupta, Shourya Chaudhary

**Affiliations:** 1 Orthopedics, Dr. D. Y. Patil Medical College, Hospital & Research Centre, Dr. D. Y. Patil Vidyapeeth (Deemed to be University), Pune, IND

**Keywords:** post operative rehab and soft tissues injuries, surgical management, traumatic finger injury, distal interphalangeal joint dislocation, flexor digitorum profundus avulsion

## Abstract

An irreducible closed dorsal dislocation of the distal interphalangeal (DIP) joint of the finger is a rare injury, often caused by factors such as the interposition of the volar plate, entrapment of the flexor digitorum profundus (FDP) tendon behind the head of the middle phalanx, or the buttonholing of the middle phalanx head through the volar plate or flexor tendon. This case report presents a rare instance of FDP avulsion combined with dorsal dislocation of the DIP joint in a 42-year-old male who sustained trauma to his right middle finger during a workplace accident. Clinical examination and imaging confirmed FDP avulsion along with dorsal dislocation of the DIP joint. Urgent surgical intervention was performed, successfully reducing and repairing the FDP tendon and stabilizing the DIP joint. Subsequent follow-up showed satisfactory functional outcomes. This case highlights the importance of prompt diagnosis and appropriate surgical management in treating complex finger injuries.

## Introduction

Finger dislocations are common hand injuries, with the distal interphalangeal (DIP) joint - a hinge joint - stabilized by both static and dynamic structures [1]. Flexor digitorum profundus (FDP) avulsion injuries, when combined with dorsal dislocation of the DIP joint, are rare and present significant diagnostic and management challenges. If not promptly recognized and treated, these injuries can lead to substantial hand function impairment [2]. In this report, we present a case of FDP avulsion with DIP dorsal dislocation in a middle-aged male, discussing the clinical presentation, diagnostic approach, surgical intervention, and postoperative outcomes.

## Case presentation

A 42-year-old right-handed male arrived at the emergency department with severe discomfort and deformity in his right fourth digit, following an industrial accident in which his finger became trapped in heavy machinery.

Examination revealed significant swelling, deformity, and inability to flex the DIP joint of the right fourth digit. Tenderness was noted over the volar aspect of the finger, and passive flexion of the DIP joint was restricted. Neurovascular examination showed intact sensation and capillary refill.

**Figure 1:**
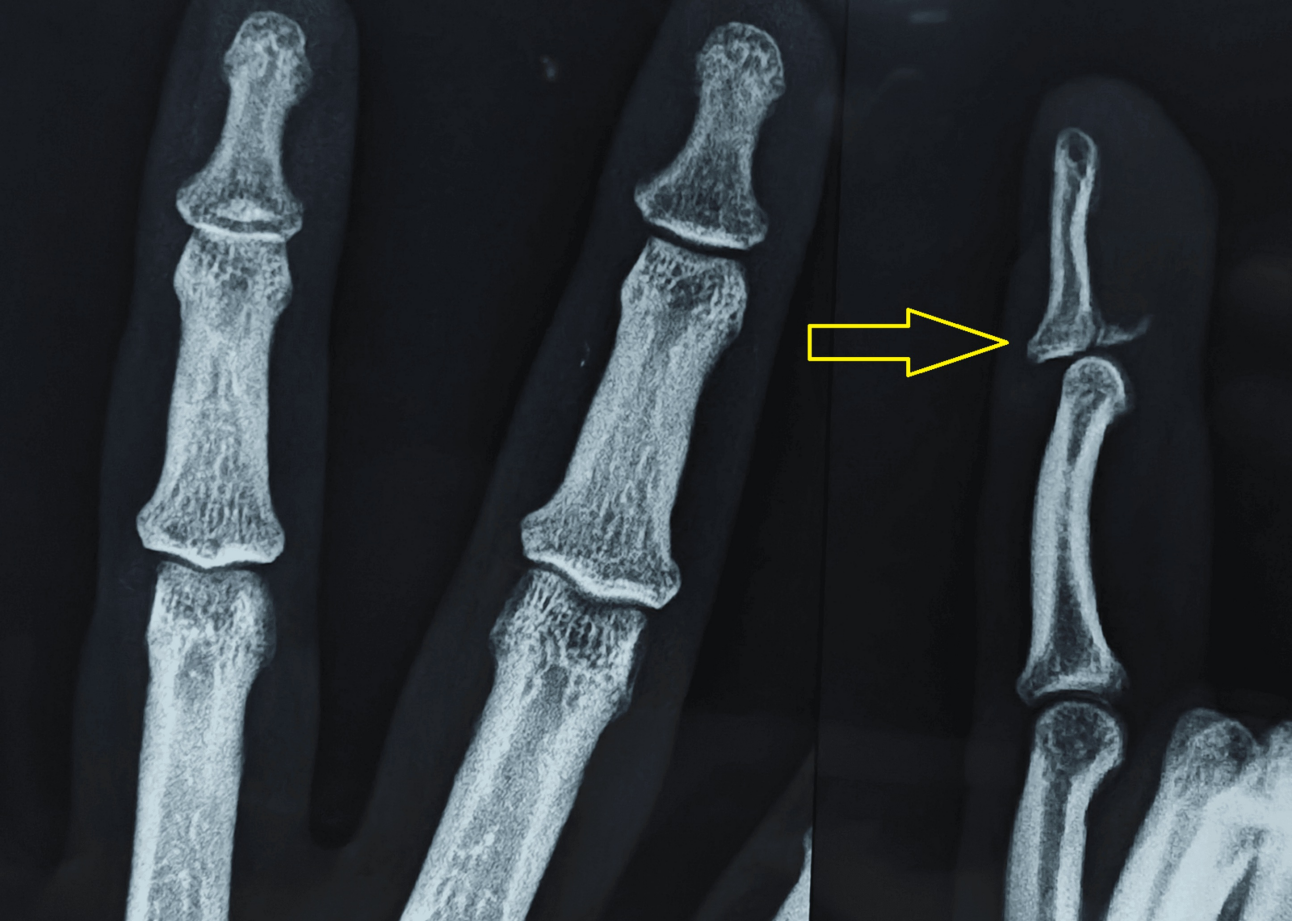
Preoperative radiograph displaying a displaced DIP joint on the lateral view (yellow arrow) DIP, distal interphalangeal

**Figure 2:**
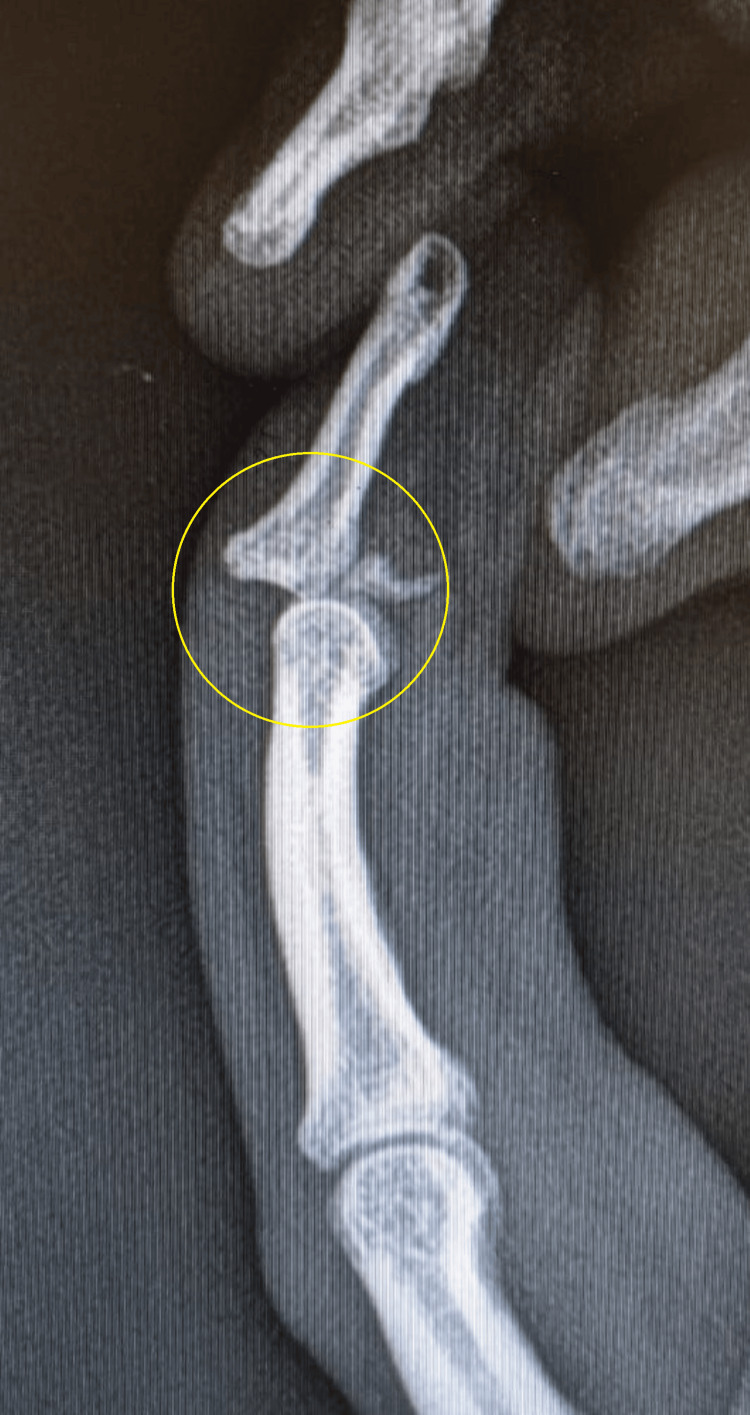
C-arm image illustrating an attempt at relocation The circle shows the DIP joint could not be reduced. DIP, distal interphalangeal

X-rays of the finger demonstrated a dorsal dislocation of the DIP joint and a fracture at the base of the distal phalanx of the fourth digit (Figure 1). An attempt at reduction under C-arm guidance proved unsatisfactory (Figure 2).

**Figure 3:**
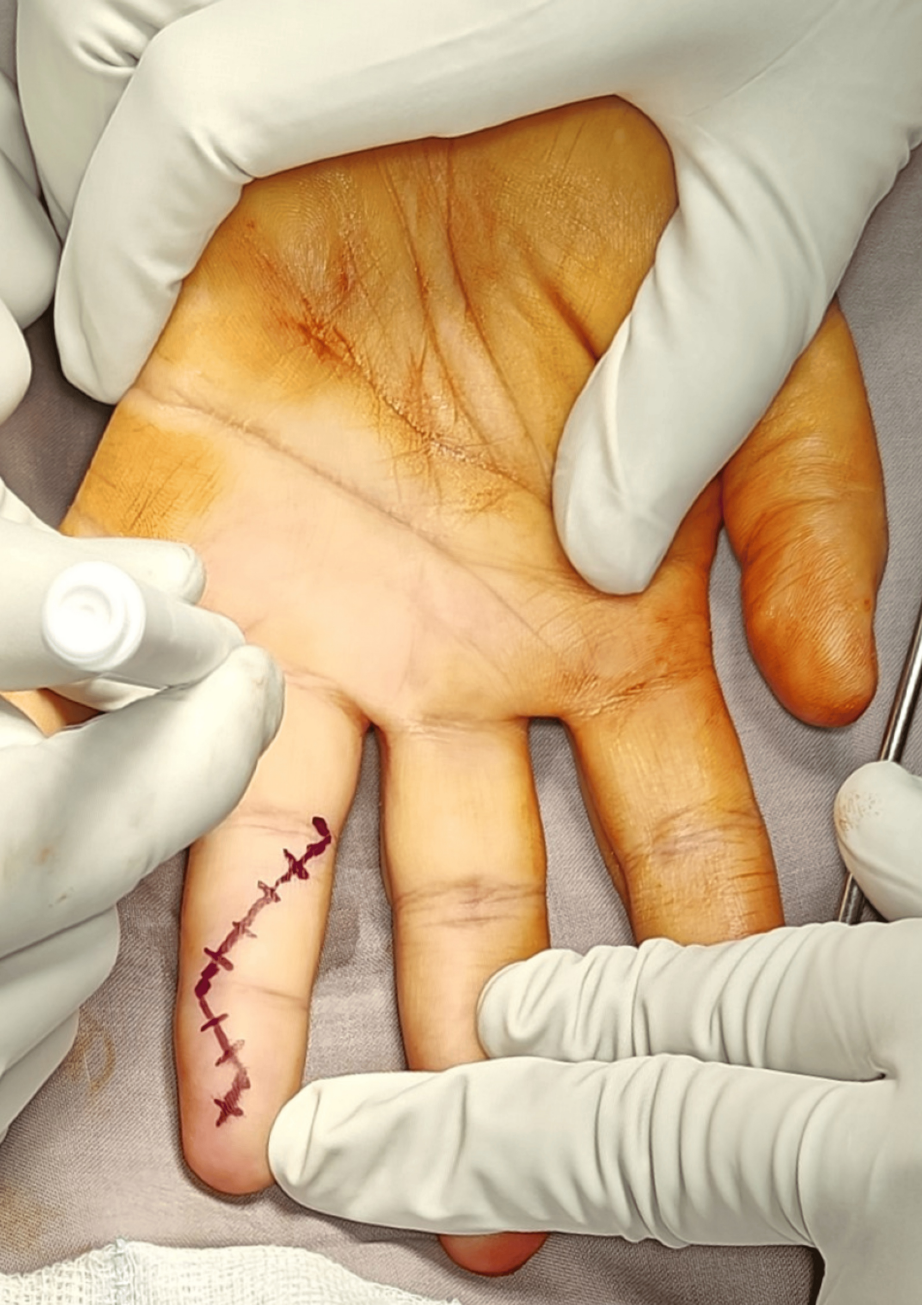
Incision marked on the volar aspect of the fourth phalanx

**Figure 4:**
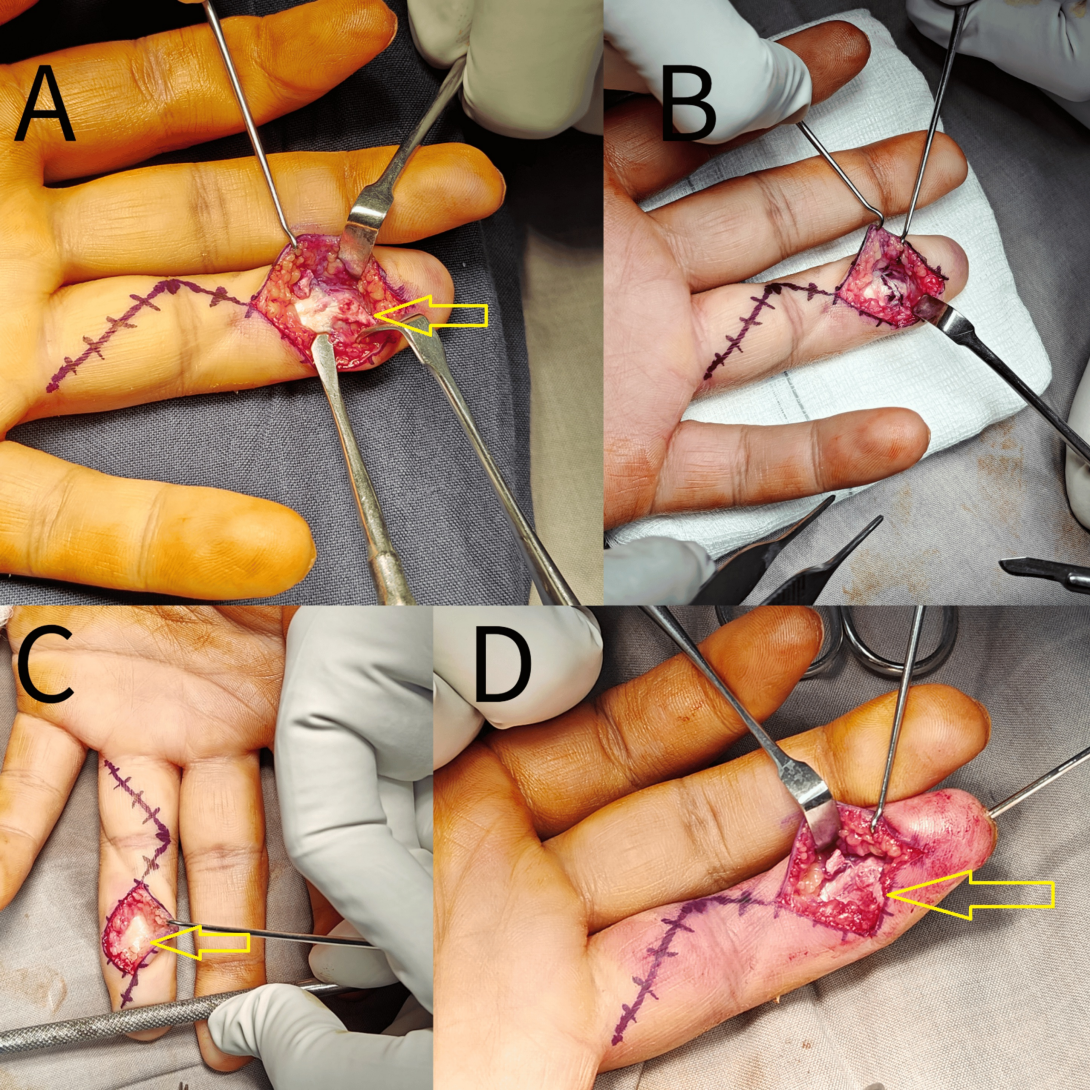
Intraoperative images showing tendon repair (A, B, C, D) The incision was used to access and repair the avulsed FDP tendon, with arrows indicating the tendon. FDP, flexor digitorum profundus

**Figure 5:**
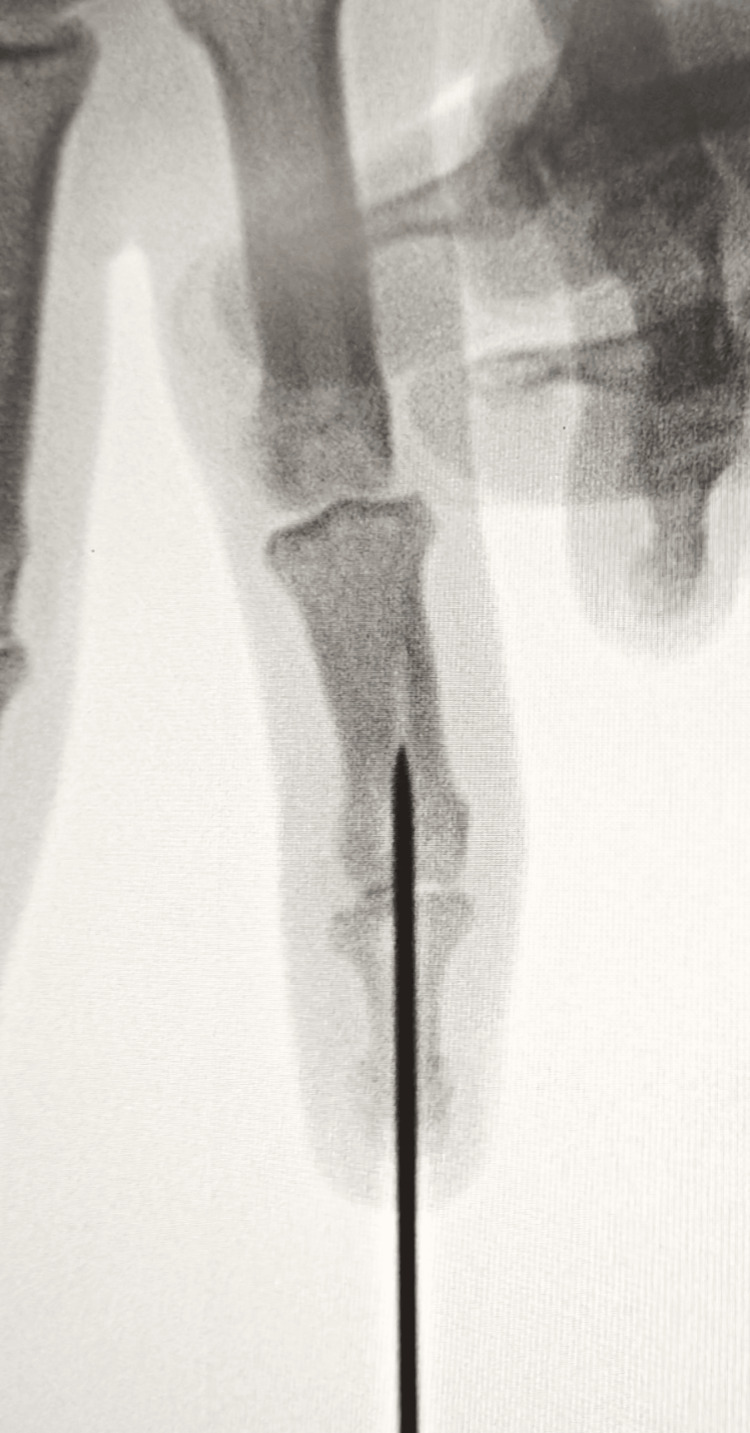
AP view of intraoperative C-arm view

**Figure 6:**
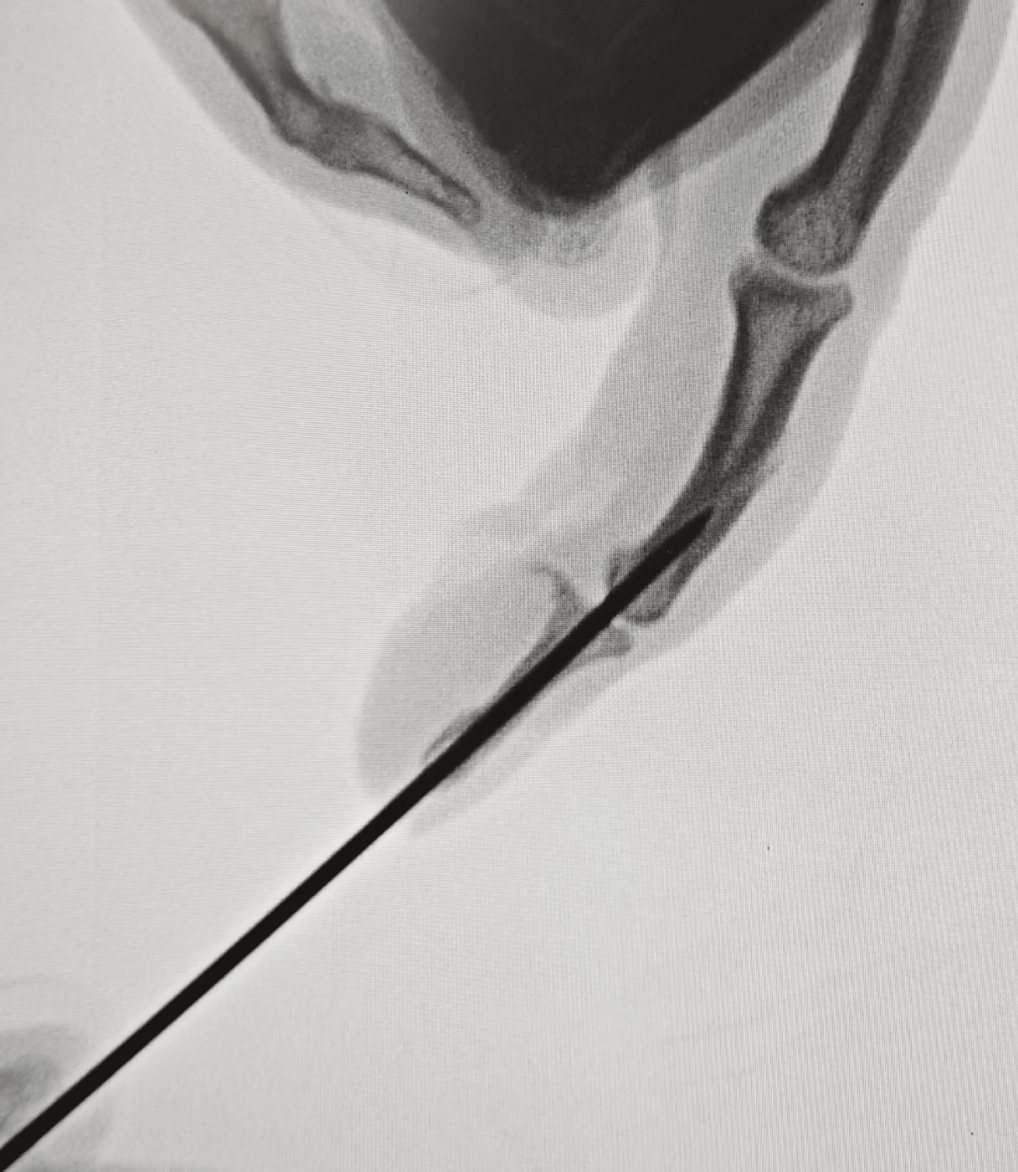
Lateral view of intraoperative C-arm view

**Figure 7:**
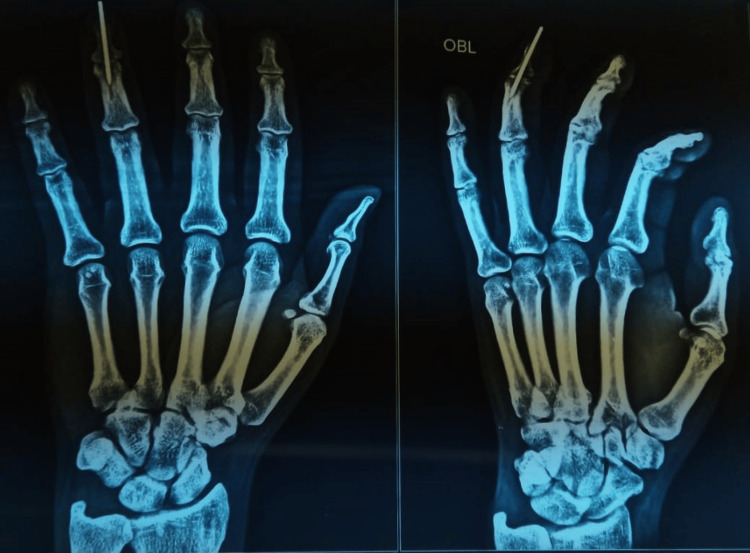
Postoperative radiograph showing AP and lateral views

**Figure 8:**
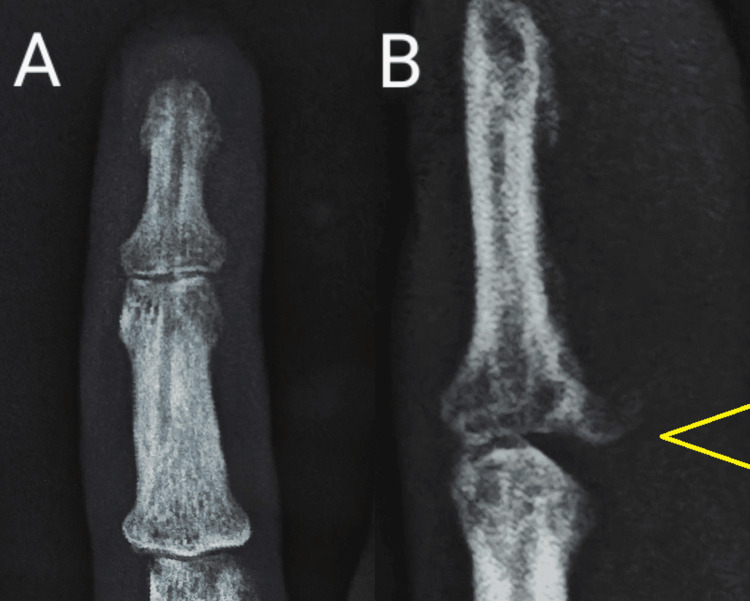
Follow-up radiograph after K-wire removal (A) AP view. (B) Lateral view.

**Figure 9:**
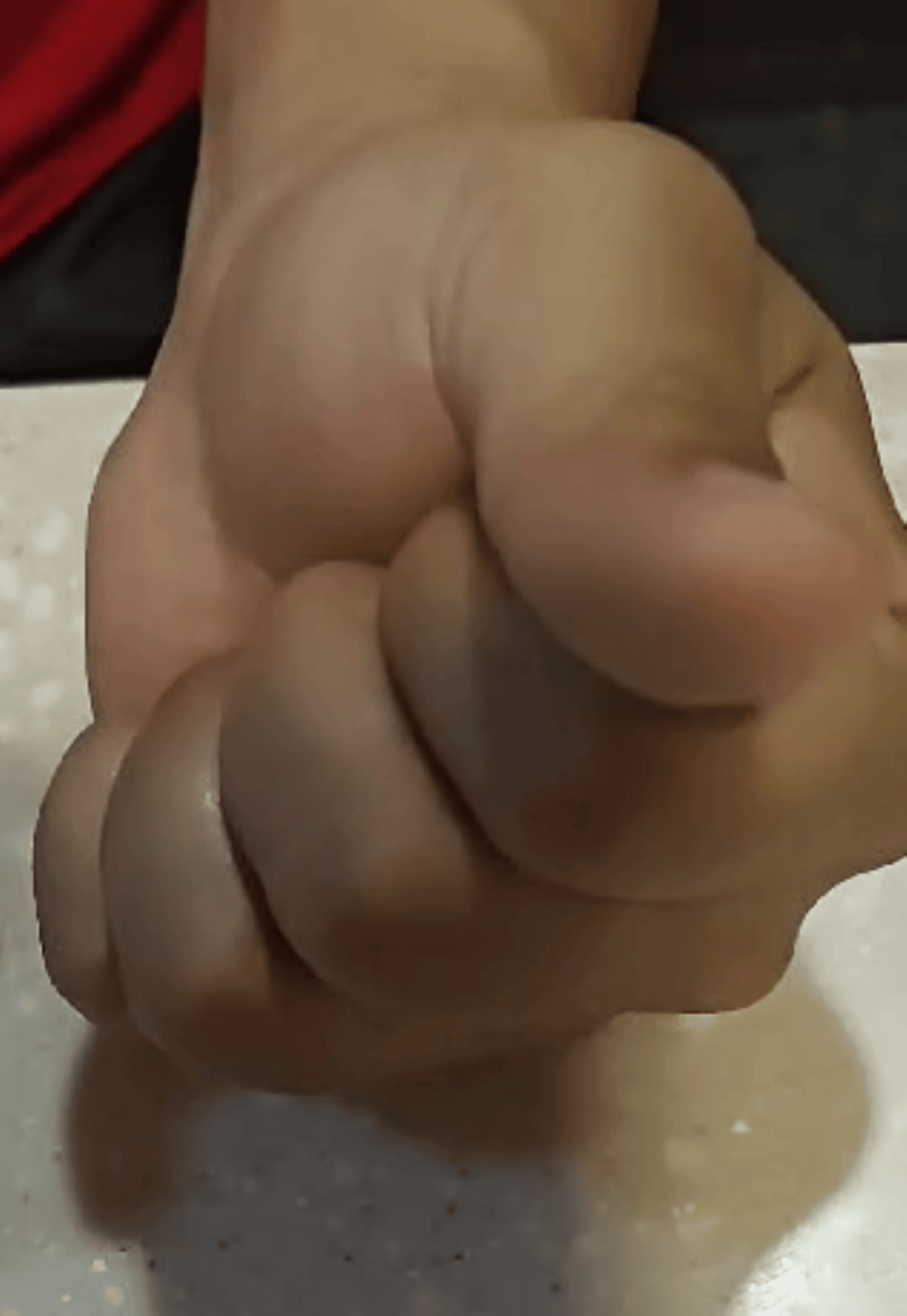
Full finger flexion achieved at three-month follow-up

Due to the irreducible nature of the joint, an associated tendon injury was suspected. The patient was scheduled for open reduction and exploration of the FDP tendon, with repair planned if necessary. After a thorough anesthesia evaluation, the patient underwent surgery under the regional block with a combined team of orthopedic surgeon, hand surgeon, and plastic surgeon. The patient was positioned supine, and the incision was marked as shown in Figure 3. During exploration, the FDP tendon was found to be avulsed and was repaired using Monocryl 4-0 (Figure 4). The DIP joint was relocated and fixed with a K-wire, which was passed from the pulp of the fourth digit to the middle phalanx, under C-arm guidance (Figure 5, Figure 6). Postoperative radiographs confirmed satisfactory reduction and fixation (Figure 7). At the six-week follow-up, the K-wire was removed, and the wound had healed completely (Figure 8). Gradual range of motion exercises were initiated, and at the three-month follow-up, the patient achieved a full range of movement, as demonstrated in Figure 9.

## Discussion

FDP avulsion injuries combined with dorsal dislocation of the DIP joint are uncommon but clinically significant. These injuries typically result from forceful hyperextension of the digit or high-energy trauma. Prompt diagnosis requires a thorough clinical evaluation and appropriate imaging studies, such as MRI and radiographs, to assess the extent of soft tissue and bony involvement.

The DIP joint is a hinge joint that allows both extension and flexion. Its stability is primarily maintained by the surrounding ligamentous structures rather than the bony anatomy. The extensor terminal tendon prevents hyperflexion, while the FDP tendon, volar plate, and collateral ligaments resist hyperextension [3]. Hyperextension, hyperflexion forces, and axial compression are the main causes of DIP joint dislocations. Typically, the reduction can be achieved with simple longitudinal traction or spontaneously. Effective reduction of a dorsally displaced DIP joint involves applying direct pressure, hyperextension, and axial traction at the base of the distal phalanx. However, irreducible dislocations are rare. Literature indicates that dorsal dislocations are more common than volar dislocations [4].

The irreducibility of the DIP joint can be attributed to several factors. An avulsed volar plate from the middle phalanx or a subluxated FDP tendon caught behind the condyle of the middle phalanx can act as an interposing mechanism. Additionally, a split in the volar plate or FDP tendon may cause buttonholing of the middle phalanx head [5]. Open and closed injuries differ in the factors contributing to irreducibility. Open dislocations typically result from FDP tendon displacement to the dorsal side of the middle phalanx condyle [6], while closed injuries often involve both entrapment and avulsion of the volar plate in the joint.

Radiographic evaluation is crucial for diagnosing and understanding the underlying cause of irreducibility. On posteroanterior X-rays, a straight dorsal dislocation may indicate buttonholing through the volar plate or involvement of the FDP tendon and volar plate interposition. Dislocation of the distal phalanx, either toward the ulnar or radial side, along with dorsal dislocation, suggests that the FDP tendon has shifted above the condyle of the middle phalanx [7].

In this case, the distal phalanx was dislocated toward the dorsal-central direction. During surgery, the avulsed volar plate was observed interposed between the two phalanges. There is some debate about the optimal surgical approach for open reduction in these injuries. While the dorsal approach simplifies joint reduction and assessment, it may impair wound healing and obscure volar structures. The volar approach was chosen in this case as it allowed for clear visualization of the trapped structures, evaluation of associated injuries such as ruptured FDP tendon and collateral ligament damage, and effective restoration of the damaged structures [6].

## Conclusions

FDP avulsion injuries with DIP dorsal dislocation are rare but require prompt recognition and surgical intervention to ensure optimal outcomes. Effective management typically involves a multidisciplinary approach, including orthopedic and hand surgeons, with careful attention to anatomical structures and functional goals. Surgical intervention is generally necessary to achieve anatomical reduction of the DIP joint and repair the avulsed FDP tendon. Treatment may involve various techniques, such as open reduction and internal fixation of associated fractures, as well as tendon repair and reconstruction, tailored to the specific injury pattern and patient factors. Long-term follow-up is crucial to evaluate functional recovery and address potential complications.
